# Electric field induced pure spin-photo current in zigzag stanene and germanene nanoribbons

**DOI:** 10.1038/s41598-022-11413-3

**Published:** 2022-05-12

**Authors:** F. Rahimi, A. Phirouznia

**Affiliations:** 1 Department of Physics, Azarbaijan Shahid Madani University, Tabriz, 53714-161 Iran; 2 Condensed Matter Computational Research Lab, Azarbaijan Shahid Madani University, Tabriz, 53714-161 Iran

**Keywords:** Nanoscience and technology, Optical properties and devices, Two-dimensional materials

## Abstract

The spin-photo current in single layer stanene and germanene under a linearly polarized light is theoretically investigated based on the tight-binding Hamiltonian combined with the nonequilibrium Green’s function at room temperature. The results show that by considering the simultaneous effect of the linear illumination and a vertical external electric field without any magnetic exchange element, pure spin-photo current without charge current is generated in two-dimensional lattices with a large intrinsic spin–orbit coupling. The necessity of enhanced spin life-time for detection of spin polarization can be explained by spin-valley locking concept. Spin-valley locking arises in buckled two-dimensional materials as a result of the large spin–orbit coupling and electric-field reversible valley spin polarization. Equal absorption of the linearly illumination at both valleys with different spin polarization, leads to pure spin-photo current injection. In addition, an acceptable photoresponsivity has been reported in a broad range of photon energy. The numerical results indicate high quantum efficiency with a maximum of nearly 83% and 50% for stanene and germanene, respectively. This work may pave theoretical reference toward design of new spin-optoelectronic devices based on satanene and germanene junctions with high performance.

## Introduction

One of the key steps towards spin-dependent optoelectronics is to produce and manipulate spin current in an efficient approach^1,2^. On the other hand, motivated by the successful isolation of graphene monolayers^3^, a lot of attention has continuously been conducted to find two-dimensional (2D) materials with new features for next-generation optoelectronics devices^4^. In this regard, the corresponding single layers honeycomb structures of other group-IV elements (Si, Ge and Sn) have been suggested theoretically^5–7^ and successfully synthesized in laboratory^8–10^. The larger bond length between heavy atoms of group-IV leads to a partial $sp^{3}$ hybridization which makes slightly buckled structure. Indeed, two triangular sub-lattices in this class of 2D materials are displaced in the out-of-plane direction with respect to each other. Different from graphene, this buckling enhances intrinsic spin–orbit coupling and introduces existence of electronic states with nontrivial topological in these materials^11^. The presence of topological phases in this type of material lead to rich physics and would possibly put forward new applications in spintronic. For example Rachel and Ezawa exhibit that edge manipulation in silicene, germanene and stanene leads to a giant magnetoresistance and perfect spin filter^12^. Furthermore, pure spin current and perfect valley filter are realized in 2D honeycomb lattices^13^. Recently, a device based on monolayer stanene was suggested that is able to generate highly spin-polarized currents up to 98$\%$^14^. Very recently it is reported fully spin-valley-polarized in zigzag stanene and germanene nanoribbons under applying a vertical electric field^15^.

In the recent years, optical properties of 2*D* monolayer structures have been also interested . Zheng et al. have theoretically suggested an X ene (X = Si, Ge, or Sn) topological transistor. Their result shows that only by tuning strength and polarization of light, the transistor can be switched between a conductive and a non-conductive state^16^. The optical response of silicene and germanene have been investigated using an electrically tunable band gap in the presence of variable doping^17^. Recently, optical properties of stanene and stanane (fully hydrogenated stanene) under the effect of strain and in the presence of spin–orbit coupling have been studied within many-body effects. Due to optical gap of stanane under strain, this material is regarded as a compelling candidate for application in optoelectronic devices such as solar cells^18^. It has been found that the optical spectra of gemanence sheet includes significant light absorption of the solar spectrum and optical response is shifted from infrared region to higher energies by applying bias voltage^19^. More importantly, it is reported that existence of excitonic insulator phase in 2D materials under the effect of small electric fields. Excitonic properties have dominant effects in computing electronic and optical features^20^.

Recently, bulk spin photovoltaic effect for generating pure spin current based on nonlinear optical theory has been investigated. The only requirement for bulk spin photovoltaic is inversion symmetry breaking. With ab initio calculations, this theory is applied to several distinct materials such as monolayer transition metal dichalcogenides, anti-ferromagnetic bilayer MnBi2Te4, and the surface of topological crystalline insulator cubic SnTe^21^. Furthermore, an efficient spin-light conversion via the Rashba and higher-order cubic Dresselhaus spin orbit interactions in ferroelectrics is predicted. Using first-principles simulations, it is demonstrated that the photo-induced pure spin current is about two orders of magnitude larger than the charge photocurrent. Interestingly, it is possible to switch the direction of spin current by an applied electric field via inverting the spin textures^22^.

The modulation of band gap and other electronic properties in nanostructures are of deep scientific interest. Interestingly, the buckled structure of 2*D* materials allows one to tune band gap via an external electric field, structural modifications (i.e., strain engineering) and chemical doping. In this respect, some studies have been performed. Using ab initio computations, it is shown that a vertical electric field is able to generate a tunable band gap in semimetallic silicene and germanene. Their results indicate that the electric field strength increases size of band gap in both systems^23^. A previous study on the effect of strain on free standing buckled germanene shows that unlike to biaxial strain, small uniaxial strain opens a direct gap at the *K* point^24^. Meanwhile, Mogulkoc et al. studied the effects of external electric and magnetic fields on electronic and transport features of corrugated stanene^25^. Using density functional theory, it is predicted that patterned boron-nitride doping creates a band gap in stanene^26^. It has been investigated that the strain engineering is a suitable approach to generate and tune the band gap in stanene lattice^27^.

Although electronic and spintronic properties of stanene under the effect of both electric field and strain have been investigated considerably, to the best of our knowledge, spin-photovoltaic properties of stanene under an electric field have not been explored yet. In this study, by employing the self-consistent nonequilibrium Green’s function (*NEGF*) approach and the tight-binding Hamiltonian model, we theoretically investigate spin-dependent optoelectronic properties of monolayer stanene zigzag nanoribbon (*SZNR*) illuminated with monochromatic linearly polarized irradiation. Photon energy range is limited to the interval $2.2\,{\text {eV}}<{\text {E}}_{{\text {ph}}}<4.1\,{\text {eV}}$. The results show that pure spin-photo current without charge current can be achieved. A pure spin current has Joule-heat-free and ultra-low-power specifications^28^. These properties cause spintronic devices as good candidates for applications in quantum computing and high speed processing^1^. Also, to examine the robustness of results over the nanoribbon width, we computed quantum efficiency for nanoribbons with different widths . The obtained results confirm that all of the nonaribbons with different size have acceptable quantum efficiency.

## Results and discussion

In the present study, it has been calculated the spin-photo current across single layer stanene zigzag nanoribbons. The incident illumination is considered monochromatic which spanning a wide frequency range with the constant intensity of $$ I_{w}=100\,\frac{{\text {kW}}}{{\text {cm}}^{2}} $$. Also, it is assumed that the electric field component of light is polarized in one given direction^29^. The linearly polarized light is shedding perpendicularly on the top of central scattering region. In performed simulations, the interaction of light with the matter is calculated using the self-consistent *NEGF* formalism. The nanodevices have been studied are stanene zigzag nanoribbons consist of constant length with $$ M = 120 $$ unit cells in scattering region. Each unit cell includes $$ N_{c}$$ atoms with *N* zigzag chains across the width of ribbon (*NSZNR*) . The central region is sandwiched between two semi-infinite left and right leads. It is supposed the same structure for the central region, the left and right electrodes. It is worth to mention that we have performed our calculations at room temperature.

**Figure 1:**
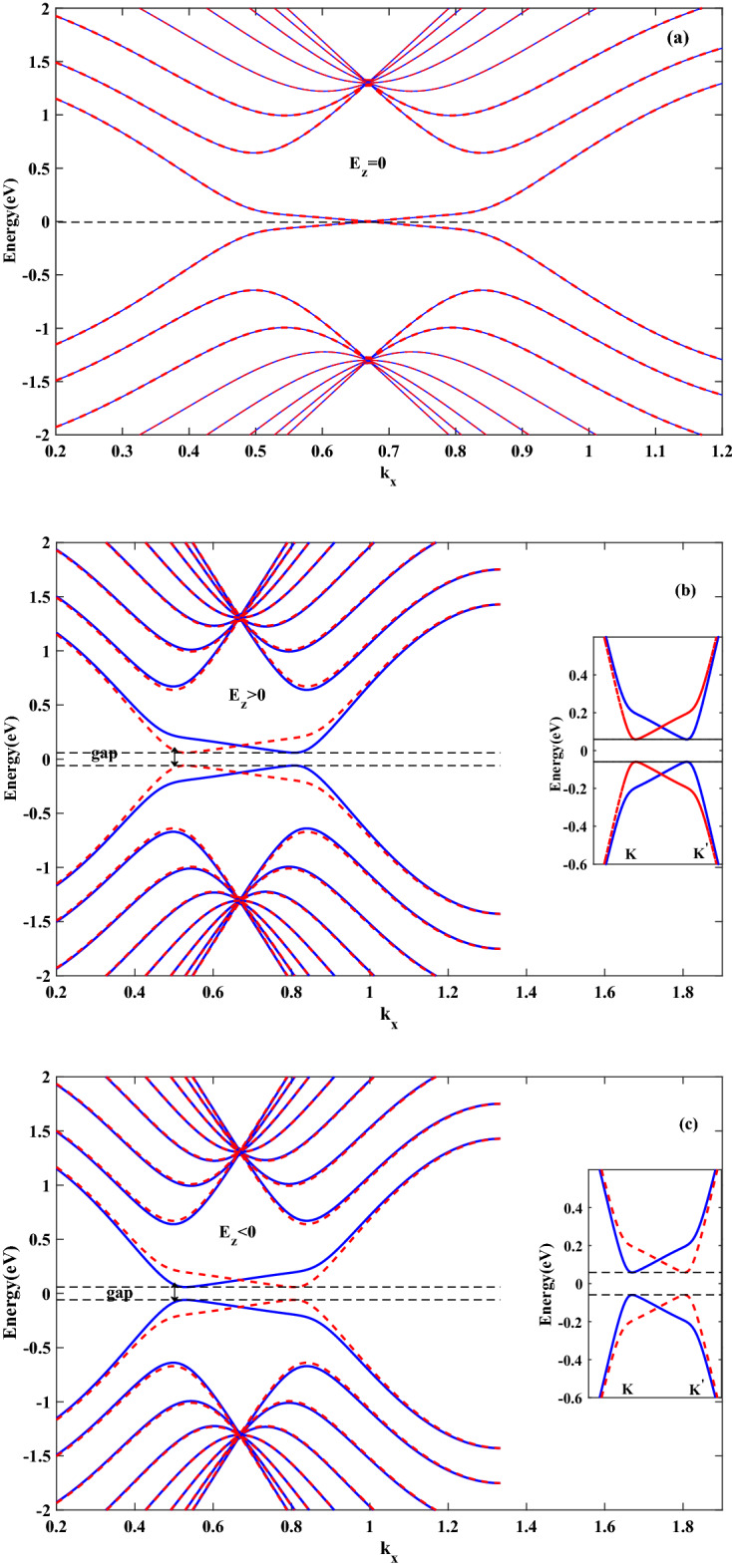
The band structure of 8*SZNR* subject to a perpendicular electric field: (**a**) with $E_{z}=0$ (**b**) with $el E_{z}=0.096t$ (**c**) with $el E_{z}=-0.096t$. Blue line denotes spin down and dashed red line denotes spin up.

In the beginning, a nanoribbon with $N_{c}=16$ atoms is considered, which is equivalent to 8*ZSNR*. To investigate the spin transport properties, firstly it is analyzed the energy electronic band structures of stanene for various strengths of $E_{z}$ shown in Fig. 1. The band structures are presented in Fig. 1 are in good agreement with a previous study^30^, guaranteeing the validity of present results. Fig. 1a–c display the band structures of *SZNR* with $E_{z}=0$, $E_{z}>0$ and $E_{z}<0$, respectively. Figure 1a shows that in the absence of vertical electric field, the band structures consist of twofold spin degeneracy. Due to the existence of large spin–orbit coupling in stanene lattice, in the presence of normal electric field, band degeneracy of spin-resolved energy levels in conduction and valence bands is split. Furthermore, finite energy band gap is produced between spin-up and spin-down energy levels (Fig. 1b,c). Also, it is obvious from these figures, switching the direction of external field will reverse the spin polarization of energy levels and, consequently, the sign of spin polarized photocurrent. Also, as Fig. 1b,c show and because of the on-site energy of Hamiltonian, it can be understood that $E_{z}$ creates approximately same displacement into the spin-down and spin-up energy levels and hence in the presence of $E_{z}$, same occupation numbers and thus same electronic features can be obtained. Physically, owing to spin splitting in the band structure (produced band gap), resulting from applied perpendicular electric field, photons of an appropriate energy are able to excite the spin-polarized carriers from valence band to conduction one, which creates spin-photo current. On the other hand, one of the intriguing properties of 2D buckled structures such as stanene and germanene is spin-valley locking. In this materials, the time reversal symmetry gives rise opposite signs of spins in two valleys, results in an effective spin-valley locking effect^31,32^. In the absence of any magnetic field, system is time reversal symmetric. As a result, spin-valley locking effect extends spin and valley relaxation times^32,33^.

**Figure 2:**
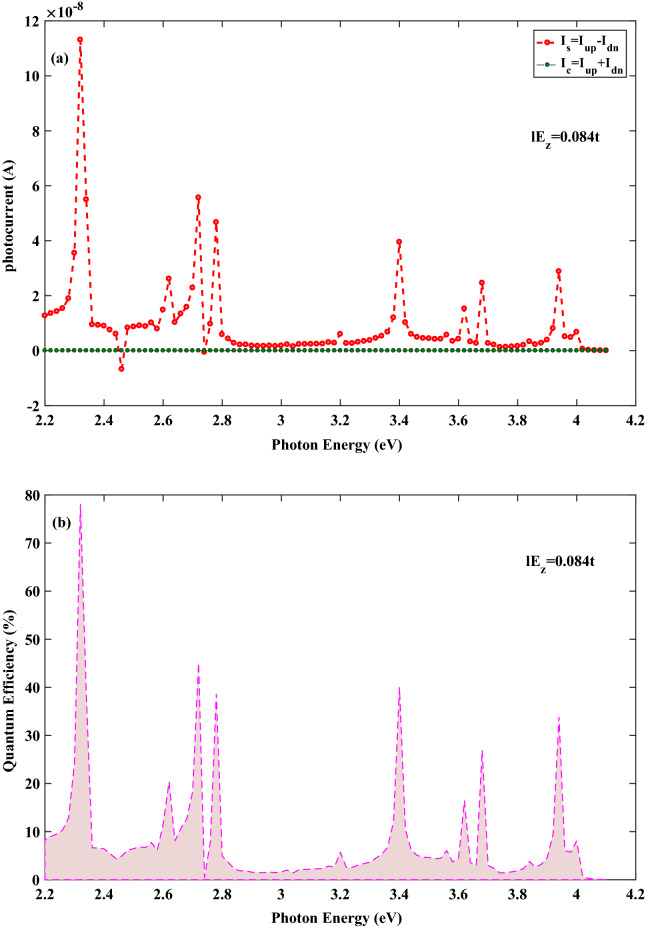
(**a**) Spin and charge photocurrent and (**b**) Quanmtum efficiency as a function of the photon energy for the spin-photovoltaic device based on 8*SZNR* under the simultaneous effect of linear illumination with $I_{w}=100\,\frac{{\text {kW}}}{{\text {cm}}^{2}}$ and $el E_{z}=0.084t$.

**Figure 3:**
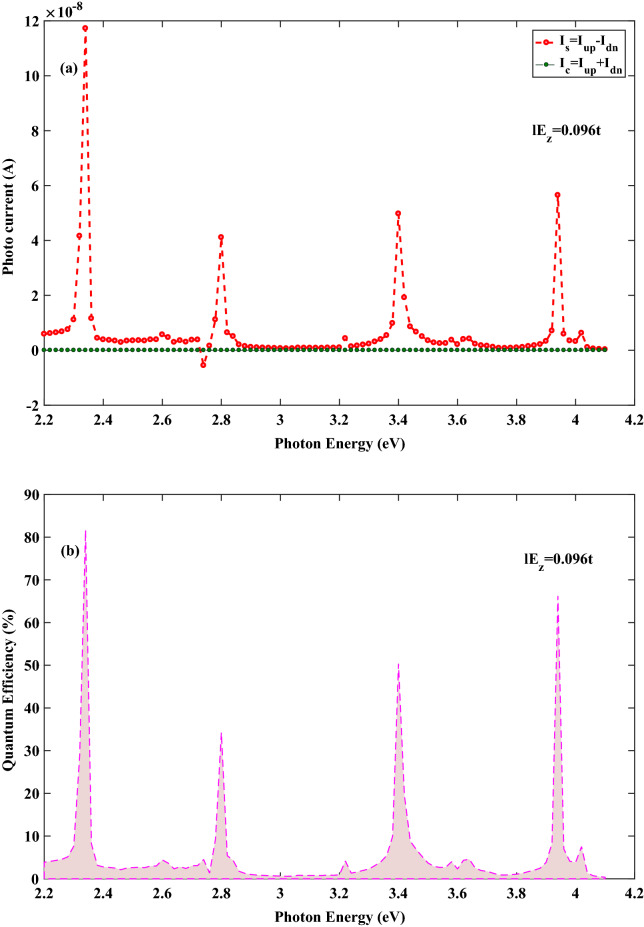
(**a**) Spin and charge photocurrent and (**b**) Quanmtum efficiency as a function of the photon energy for the spin-photovoltaic device based on 8*SZNR* under the simultaneous effect of linear illumination with $I_{w}=100\,\frac{{\text {kW}}}{{\text {cm}}^{2}}$ and $el E_{z}=0.096t$.

Now, we inspect the spin-photo current in stanene lattice which is generated when the central region is irradiated with the linearly polarized electromagnetic wave where placed under the effect of vertical electric field. The spin current is defined as $$ I_{s}=I_{up} -I_{dn}$$ and the charge current is obtained by $$ I_{c}=I_{up} +I_{dn}$$ , where $$ I_{up}(I_{dn}) $$ denotes spin-up (spin-down) current. Calculations show that $$ I_{s}\ne \,0$$ and $$ I_{c}=0$$, which means the generation of pure spin-photo current in stanene nanoribbon. It is well known that the ultimate goal in spintronics is to use pure spin current for the sake of minimizing the Joule heating^34^. Figures 2a and 3a represent spin and charge current as function of photon energy for 8*SZNR* with $$ el E_{z}=0.084t $$ and $$ el E_{z}=0.096t $$, respectively. For the photon energy between 2.2 and $$ 4.1\,{\text {eV}} $$, we obtain $$ |I_{up}|=|I_{dn}| $$, resulting pure spin current without charge current. As presented explicitly in the inset of Fig. 1b, for stanene lattice, the spin-down band of the *K* valley is far from Fermi energy and hence it has negligible contribution in transport. Therefore, spin-photo current in the *K* valley is produced by spin-up photo-excited carries . Contrastingly, in the $$K^{'}$$ valley, the most contribution in the generated spin-photo current is devoted to the spin-down carriers. Meanwhile, it should be noted that the magnitude of electric field induced band gap is equal for two valleys. Accordingly, the absorption of photon is nearly equal for the spin-up and spin-down carries in the *K* and the $$ K^{'} $$ valleys, respectively. Thus, same population of opposite spin-polarized carriers can be obtained in each of the valleys. Since impurities or the electron-phonon interaction are not included in this study, spin relaxation mechanism and hence spin flipping which is accompanied with momentum scattering that may come from Elliott–Yafet or any other spin relaxation mechanisms is neglected^35^. Consequently, the acceptable optical transitions only take place between the sub-bands with an equal spin by shining a linearly polarized illumination. The population balance between the *K* and the $$ K^{'} $$ ($$ K^{'}=-K $$ ) valleys with opposite spin polarization leads to spin-up and spin-down currents with same absolute value but in opposite directions. In other words, by considering positive momentum for spin-up photo excited carriers in the *K* valley, spin-down photo excited carries have negative momentum in the $$ K^{'} $$ valley ($$ K^{'}=-K $$). Therefore, different spin polarized carries with same population move in opposite directions, which can result in non-equilibrium pure spin-photo current with zero charge current. However, it should be noted the produced current is obtained only in the presence of incident illumination. Thus, this pure spin current is attributed to the non-equilibrium state. Meanwhile, in the presence of incident illumination, the non-equilibrium population is equal for spin-up and spin-down states in different valleys.

Figures 2b and 3b demonstrate quantum efficiency for 8*SZNR* with $el E_{z}=0.084t$ and $el E_{z}=0.096t$, respectively. One of the key parameters to access the spin-dependent optical performance of nanodevice is quantum efficiency and it is defined as

1 $$\begin{aligned} \eta _{\sigma }=\frac{E_{ph}\,I_{ph,\sigma }}{e\,A_{D}\,I_{w}}\times 100 \%, \; (\sigma =up, down) \end{aligned}$$

where $A_{D}=L_{ch}\,W_{ch}$ and $E_{ph}$ are cross section of central channel and photon energy, respectively. In this case, because of equally generated down and up spin currents given by $|I_{up}|=|I_{dn}|$, results in $\eta _{up}=\eta _{dn}$ and therefore only quantum efficiency is displayed for spin-up in figures. As shown in Figs. 2b and 3b, quantum efficiency changes in the photon energy range considerably. These figures show that increasing the electric field strength effects on quantum efficiency in the whole energy range. Also, Figs. 2b and 3b reveal that the highest peaks of quantum efficiency with values of nearly 78% and 82% corresponding to the most probable optical transitions in SZNR channel, occur at $E_{ph}=2.3\,{\text {eV}}$, with $el E_{z}=0.084t$ and $el E_{z}=0.096t$, respectively. The positions of the highest peaks of quantum efficiency are almost constant for different values of the field. However, the number of peaks varies by varying the strength of the electrical field. Although the obtained quantum efficiency of optical absorption peaks with $el E_{z}=0.096t$ are higher than the case $el E_{z}=0.084t$ , generally, $el E_{z}=0.084t$ generates higher quantum efficiency in the given photon energy range. Furthermore, the least probable transitions for different strength of electrical field takes place for the photon energy range at $2.9\,{\text {eV}}<E_{ph}<3.1\,{\text {eV}}$. It is worthy to mention that the optical transition dependents on not only appropriate photon energy, but also dependents on some other factors such as optical transition selection rules. These selection rules may be acquired satisfactorily for a suitable photon energy. Furthermore, the energy bands are not fully spin-polarized and spin mixing is possible. This spin-mixing could result in new selection rules and since spin-mixing is not the same for different energies^35^, so, it is not possible to discuss about optical transition only by photon energy.

**Figure 4:**
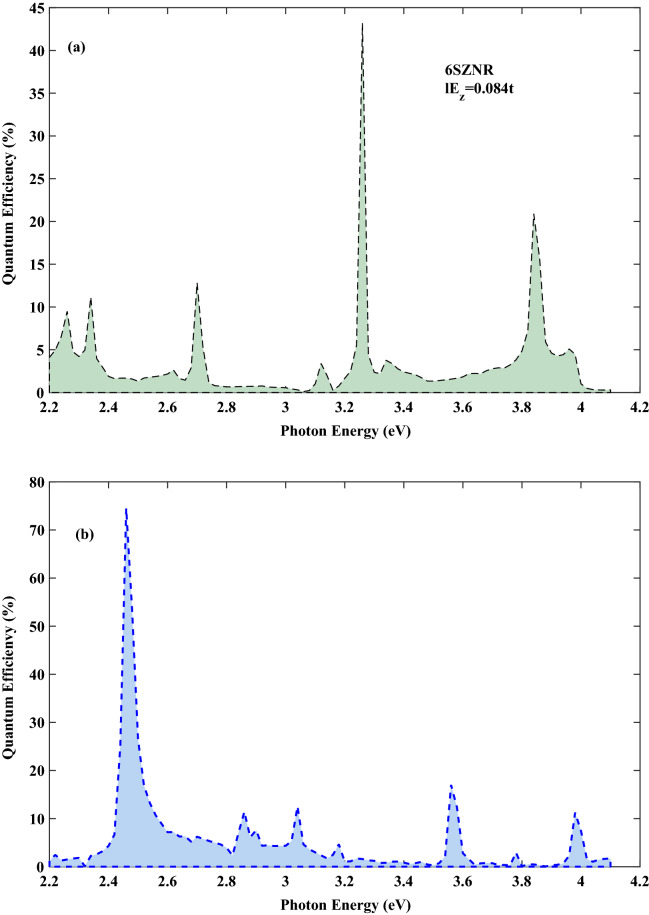
Quanmtum efficiency as a function of the photon under the simultaneous effect of linear illumination with $I_{w}=100\,\frac{{\text {kW}}}{{\text {cm}}^{2}}$ (**a**) for the spin-photovoltaic device based on 6*SZNR* with $el E_{z}=0.084t$ and (**b**) for the spin-photovoltaic device based on 10*SZNR* with $el E_{z}=0.089t$.

In the following, quantum efficiency for varies size of *SZNRs* with different widths has been computed. The results indicate that for very narrow widths of *SZNRs* ($$6<N<18 $$) and different strengths of $$ E_{z} $$ as the width of ribbon increases, the energy band gap for both spin states decreases. Therefore, the optical response of *SZNR* could change by tuning the width of nanoribbon. In Fig. 4a quantum efficiency as function of photon energy with $$ el E_{z}=0.084t $$ is computed for 6*SZNR*. Maximum quantum efficiency is about 43% at $$ E_{ph}=3.3\,{\text {eV}}$$. Also, the least probable transitions is occurred for photon energy range between 2.9 and $$3.1 \,{\text {eV}}$$ as before reported above for 8*SZNR*. In Fig. 4b quantum efficiency is displayed for 10*SZNR* with $$ el E_{z}=0.089t $$. Evidently, the most probable optical absorption occurs for photon energy interval $$2.4 \,{\text {eV}}<E_{ph}<2.6 \,{\text {eV}}$$ . Moreover 10*SZNR* indicates a high quantum efficiency with a maximum of approximately 74% .

In addition to the vertical electric field, the influence of an in-plane transverse electric field on the electronic band structure of SZNRs has been studied. The obtained results reveal that electric field in the transverse direction exerts a similar effect on the band structure of nonaribbon. Because atoms in the edges of SZNR are a member of two different sublattices, thus in the presence of an in-plane electric filed they are exposed to various potentials. On the other hand, due to the buckled structure of stanene lattice, atoms at zigzag edges of stanene lattice are located on different heights and therefore they experience various potentials under the vertical electric filed. Thereby, one can expect similar optoelectronic response under applying both vertical and in-plane electric fields.

**Figure 5:**
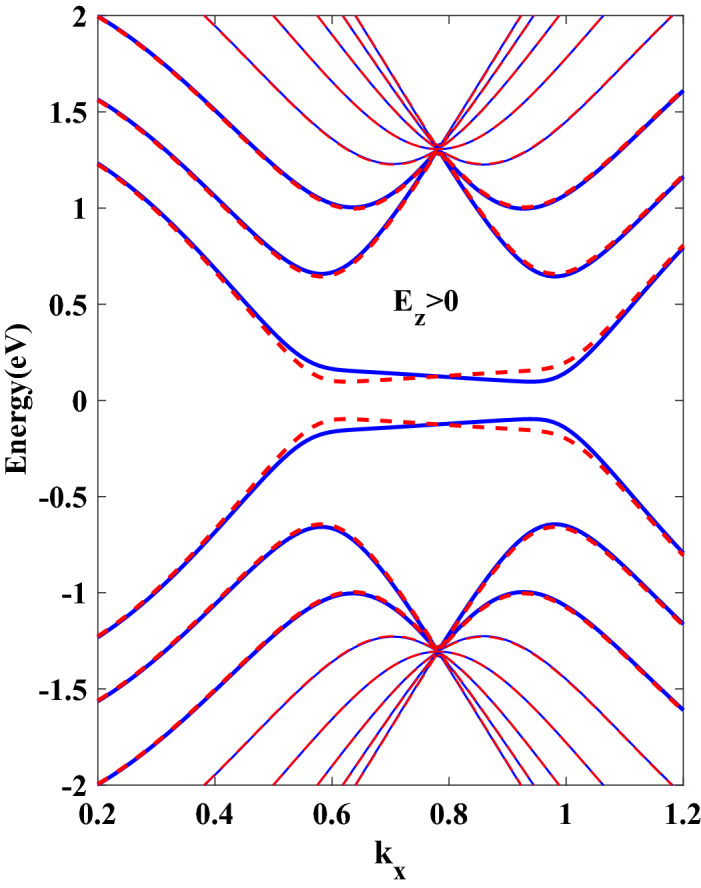
The band structure of 8*ZGNR* subject to a perpendicular electric field with $el E_{z}=0.096t$ . Blue line denotes spin down and dashed red line denotes spin up.

**Figure 6:**
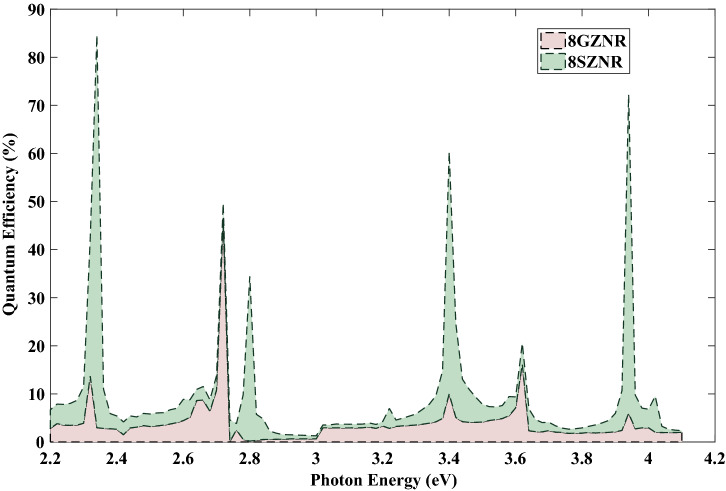
Quanmtum efficiency as a function of the photon under the simultaneous effect of linear illumination with $I_{w}=100\,\frac{{\text {kW}}}{{\text {cm}}^{2}}$ and $el E_{z}=0.096t$.

Finally, spin transport in germanene zigzag nanoribbon with $N=8$ zigzag chain across the width of ribbon (8*GZNR*) under the simultaneous effect of linear illumination with $I_{w}=100\,\frac{{\text {kW}}}{{\text {cm}}^{2}}$ and $el E_{z}=0.096t$ is investigated. To this end, we first describe the energy band structures of germanene nanoribbon, displayed in Fig. 5 and computed in the framework of the tight-binding model. The tight-binding parameters for germanene are $t= 1.3\,{\text {eV}}$, $\lambda _{so}=0.043\,{\text {eV}}$ and the buckling height is $\ell =0.33\,\mathring{A}$^36^. By comparing this figure with Fig. 1b, it is obvious that the band gap of 8*ZGNR* is larger than 8*ZSNR*, such that the calculated band gap for 8*ZGNR* is equal $0.194\,{\text {eV}}$ and for 8*ZSNR* is $0.118\,{\text {eV}}$. In the case of *GZNR* similar to *SZNR*, pure spin current without charge current is obtained, again. In order to compare the optical performance of spin photovoltaic device based on GZNR and SZNR, spin-dependent quantum efficiency of both nanoribbons for spin up state versus photon energy under the effect of vertical electric field are displayed in Fig. 6. From this figure one can see that, although the location of some spin-dependent optical absorptions is nearly constant for both ribbons but the height of spin-dependent optical absorptions is reduced considerably. For example, the first quantum efficiency peak for *GZNR* is about 82% , while for *SZNR* is about 11% . In general, according to the calculated band gap, it can be said that under same conditions of light intensity and field strength, the spin-dependent quantum efficiency of *SZNR* is higher than that of germanene.

## Method

The spin-functionalized electronic and optical properties of zigzag stanene nanoribbons are computed by means of the tight-binding Hamiltonian with the NEGF formalism. Total Hamiltonian of the nanodevice is described by:

2 $$\begin{aligned} H_{T}=H_{L}+H_{R}+H_{C}+H_{CL}+H_{CR} \end{aligned}$$

where the first two contributions in Eq. (2) are the Hamiltonian of the semi-infinite left and right leads, respectively. $$ H_{LC} $$ and $$ H_{RC} $$ describe the coupling between the scattering region and the left and right leads. $$ H_{C}=H_{0}+H_{e-ph}$$ represents the Hamiltonian of scattering region. $$ H_{0} $$ is the tight-binding approximation of a stanene nanoribbon reads as followes^36–38^:

3 $$ \begin{aligned}   H_{{0,\sigma }}  &  =  - t\sum\limits_{{\langle {\kern 1pt} i,j\rangle ,\alpha }} {c_{{i\alpha }}^{\dag } } c_{{j\alpha }}  + i\frac{{\lambda _{{so}} }}{{3\sqrt 3 }}\sum\limits_{{\langle \langle {\kern 1pt} i,j\rangle \rangle ,\alpha ,\beta }} {\nu _{{ij}} } c_{{i\alpha }}^{\dag } (\sigma _{z} )_{{\alpha \beta }} c_{{j\beta }}  \\     & \quad  + e\ell E_{z} \sum\limits_{{i,\alpha }} {\xi _{i} } c_{{i\alpha }}^{\dag } c_{{i\alpha }}  + H.c. \\  \end{aligned}  $$

$$ c_{i\alpha }(c^{\dagger }_{i\alpha }) $$ annihilates (creates) an electron with spin polarization $$ \alpha $$ at atom *i*, and $$ <i,j> $$ / $$<<i,j>> $$ run over all the nearest and the next nearest neighbor hopping sites, respectively. The first term $$ \varpropto t $$ indicates the nearest-neighbor hopping and its value is equal to 1.3 eV. The second term $$ \varpropto \lambda _{so}=0.1\,{\text {eV}}  $$ indicates the effective intrinsic spin–orbit interaction (SOI) term. $$ {\varvec{\vec{\sigma }}} = (\sigma _{x} ,\sigma _{y} ,\sigma _{z} ) $$ is the Pauli’s spin matrix. $$ \nu _{ij}=-1(+1) $$ for clockwise (anticlockwise) next-nearest-neighboring hopping. $$ \xi _{i} $$ is equal to $$ +1(-1) $$ for the upper (lower) sublattice. The fourth contribution denotes the effect of a perpendicular electric field $$ E_{z} $$ and generates voltage difference of $$ 2e\ell E_{z} $$ between the upper and lower sublattices. Also, $$\ell =0.4\,\mathring{A}$$ is the buckling height. Strength of the first and the second Rashba SOIs i.e., extrinsic and intrinsic interactions, are negligible compared to the intrinsic SOI $$ \lambda _{so} $$ and therefore we have omitted them in calculations. The first Rashba SOI associated with the nearest neighbor hopping which is induced by an external electric field and therefore it can be manipulated and even set to zero without use any type of approximations. The intrinsic Rashba SOI arising from the second-nearest-neighbor hopping terms which is very weak in comparison with the intrinsic SOI $$ \lambda _{so} $$^39^. In this article, the common nearest neighbor tight-binding Hamiltonian model of hexagonal lattices or graphene-based structures is used for studying spin-dependent optoelectronics properties of stanene and germanene lattices, in which the contribution of $$ p_{x} $$ and $$ p_{y} $$ orbitals is not considered. It should be noted that by increasing energy range, the contribution of $$ p_{x} $$ and $$ p_{y} $$ orbitals may modify in the band structure, the contribution of these orbitals will also increase the spin–orbit interaction. In fact, this simplification neglects the contribution of these orbitals in Hamiltonian. It can be said that the obtained results may be modified by considering the contribution of these orbitals^40^.

Term $H_{e-ph}=(\frac{e}{m})\, {\vec{\mathbf{A}}} . {\vec{\mathbf{P}}}$ is the perturbation Hamiltonian for the electron-photon scattering, where *m* is electron mass, ${\vec{\mathbf{A}}}$ and ${\vec{\mathbf{P}}}$ are the electromagnetic vector potential and the electronic momentum, respectively^29,41,42^.

After calculating the Hamiltonian of the scattering region and the left and right leads, the retarded Green’s function of system, in the presence of illumination, can be written as:

4 $$\begin{aligned}&G_{\sigma }(E)=[(E+i\eta )I-H_{0,\sigma }-\Sigma _{L,\sigma }-\Sigma _{R,\sigma }-\Sigma _{ph,\sigma }]^{-1} \end{aligned}$$

where $\eta$ and *I* are infinitesimal broadening and identity matrix, respectively. $\Sigma _{L(R),\sigma }$ represents the retarded self-energy of the left( right) electrode which is calculated using the Sancho iterative method^43,44^. In Eq. (4), $\Sigma _{ph,\sigma }$ is the self-energy of electron–photon scattering which is expressed as:

5 $$\begin{aligned} \Sigma _{ph,\sigma }=\frac{-i}{2}[\Sigma _{ph,\sigma }^{<}(E)-\Sigma _{ph,\sigma }^{>}(E)] \end{aligned}$$

The lesser and greater self-energy of the electron-photon interaction can be written as:

6 $$\begin{aligned} \Sigma _{ph,\sigma }^{\gtrless }(E)=(N_{ph}+1)MG_{\sigma }^{\gtrless }(E^{\mp })M+N_{ph} MG_{\sigma }^{\gtrless }(E^{\pm })M \end{aligned}$$

where $E^{\pm }=E\pm \hslash \,\omega$ and $N_{ph}$ is the number of photon with energy $\hslash \,\omega$^45^. Also, *M* contains the electron–photon interaction and matrix elements of *M* are given by:

7 $$\begin{aligned} M_{lm}=\langle \,1| {\vec{\mathbf{P}}} .\,A_{0}\,\hat{e_{p}}|\,m\rangle \end{aligned}$$

where, $$ A_{0} $$ shows amplitude of $$  {\vec{\mathbf{A}}}  $$ and $$ \hat{e_{p}} $$ is direction of light polarization. $$ G^{<} $$ and $$G^{>} $$ are the lesser and greater Green function. They are defined by employing the Keldysh equation^46^:

8 $$\begin{aligned} G_{\sigma }^{<}(E)=G_{\sigma }(E)[\Gamma _{L,\sigma }(E)\,f_{L}(E)+\Gamma _{R,\sigma }(E)\,f_{R}(E)+\Sigma _{ph,\sigma }^{<}]G_{\sigma }^{\dagger }(E) \end{aligned}$$

and

9 $$\begin{aligned} G_{\sigma }^{>}(E)=G_{\sigma }(E)[\Gamma _{L,\sigma }(E)\,f_{L}(E)+\Gamma _{R,\sigma }(E)\,f_{R}(E)+\Sigma _{ph,\sigma }^{>}]G_{\sigma }^{\dagger }(E) \end{aligned}$$

In Eqs. (8) and (9), $f_{L}(_{R})$ exhibits the left (right) Fermi function. $\Gamma _{L(R),\sigma }= i(\Sigma _{L(R),\sigma }-\Sigma _{L(R),\sigma }^{\dagger })$, represents the broadening functions of the left (right) electrode. Finally, after computing the electron–photon self-energy by solving Eqs. (5)–(9) self-consistently, spin-dependent photocurrent across the ribbon can be written as^47^

10 $$\begin{aligned} I_{ph,\sigma }=\dfrac{2e}{h}\int {dE} Tr\,[G_{(1,1)\sigma }^{>}(E)\Gamma _{L,\sigma }(E)\,f_{L}(E)-G_{(1,1)\sigma }^{<}(E)\Gamma _{L,\sigma }(E)\,(1-f_{L}(E))] \end{aligned}$$

where *h* is the Planck’s constant and *e* is the elementary charge. $G_{(1,1),\sigma }^{>}(G_{(1,1),\sigma }^{<})$ is the first blocks of the hole (electron) correlation functions.

## Concluding remarks

In this research, a way of generating a non-equilibrium pure spin current in zigzag stanene and germanene nanoribbons with a strong intrinsic spin–orbit interaction are proposed theoretically. By employing the NEGF formalism and the tight-binding Hamiltonian, the photoresponsivity of spin-photovoltaic device under the simultaneous effect of normal electric field and linearly polarized irradiation is simulated. Twofold band degeneracy between spin-up and spin-down states in conduction and valence bands is split due to the combination effect of electric field and large spin–orbit of these lattices. The spin splitting of energy levels leads to spin-dependent absorption. The symmetry of band gap energy at the *K* and the $K^{'}$ valleys gives rise to equal absorption of illumination and consequently, non-equilibrium spin population balance in these valleys. Equal occupation numbers between the *K* and the $K^{'}$ ($K^{'}=-K$) valleys creates an equal spin polarized photocurrent for spin-up and spin down components with identical absolute value but opposite directions in the whole range of photon energy. The reversal of external electric field direction changes role of spin states in valleys and thus sign of spin polarized photocurrent. Also, robustness of spin-dependent behavior over the nanoribbon width was studied.
